# Harvard Odontological Society

**Published:** 1894-10

**Authors:** 

**Affiliations:** Harvard Odontological Society


### HARVARD ODONTOLOGICAL SOCIETY.

A REGULAR meeting of the Harvard Odontological Society was held April 26, 1894, President Eddy in the chair.

### DISCUSSION ON USING THE MATRIX.

*President Eddy.*—A number of the members present were with us this afternoon at the clinic given by Dr. Werner illustrating the putting on of matrices. Perhaps some of the gentlemen would like to express their opinions on this interesting subject. I will first call on Dr. Werner.

*Dr. Werner.*—To talk intelligently on a clinic after it is over is difficult; that is one of its disadvantages. A clinic is pre-eminently an event to be present at in order to appreciate its value. I was asked to bring a few things that we used this afternoon for the benefit of those who were not present.

The matrix that seems to answer the greatest number of cases is a thin, cold-rolled steel, such as may be found in any hardware store. I get large strips, twelve to fifteen inches long and four to six inches wide. The very thinnest will please you best, as it is very elastic and can be concaved readily, and you can cut it in strips varying in width. Try one of those little slips and fit to the length that you want, cut off a little piece with your scissors, concave it with a bayonet-shaped instrument, and you have a little piece of steel concaved to the shape that your filling is desired to take. Then adjust it at the cervical wall with a little wedge, gutta-percha, or the Perry separator,—either one or all three, as the case may seem to require. Just in proportion as you are skilled in putting on the matrix, will you lighten your labors in the filling of approximal surfaces. Ten minutes is a long time to spend in making and adjusting a matrix, and after the matrix is on, an hour to an hour and a half at the most is sufficient to fill a very extensive surface of bicuspid or molar; the finishing can be done in half an hour, so that an approximal surface in a bicuspid or molar can be filled with gold in two hours, while with any other system it takes three hours, and you have not such a condensed gold filling. I believe thoroughly in gold. With gutta-percha and the cements we are simply patching over for six months, and when the patient comes back those teeth are worse to fill than ever. Wedge the teeth completely apart, no matter how long it takes; then fill the tooth firmly and you are done with it.

Here is a model that will demonstrate the kind of surfaces that are so difficult to reach. I am going to show the models of this case filled in contour later on. I will also pass around a few of the instruments that I use. These pluggers are taken from the Darby-Perry system, but enlarged and thickened at the shank for non-cohesive work. You can have tin and gold at the cervical wall in very difficult cases, and in cases that go under the gum you can fix and adjust your matrix before you put on your rubber dam. I have never seen a matrix put on by any one, but I use it invariably for all approximal surfaces that have any opening through the crown, and all naturally large decayed surfaces have. With the matrix it is not necessary to have an undercut at the weakest part of the tooth. You put your non-cohesive in and hold it there with an instrument, packing firmly. It will not hurt, because the tooth is supported by the matrix, and when you get a good foundation then finish with rolled cohesive gold, such as Williams has in high numbers,—say No. 40 or No. 60,—burnish thoroughly, and you have an ideal contour filling.

*Dr. Clapp.*—Mr. President, it seems to me that we receive great benefit from these clinics. The operation performed this afternoon was artistic, scientific, and practical in every particular, and it shows that contour work can be rapidly and well done. I must say that I have great respect and admiration for contour work.

*President Eddy.*—Under the head of Presentation of Specimens, I shall call upon Dr. P. W. Moriarity, who has a patient here in waiting.

Adjourned.

HENRY L. UPHAM, D.M.D.,
*Editor Harvard Odontological Society.*

